# Genetic Association of *PTPN22* Polymorphisms with Autoimmune Hepatitis and Primary Biliary Cholangitis in Japan

**DOI:** 10.1038/srep29770

**Published:** 2016-07-11

**Authors:** Takeji Umemura, Satoru Joshita, Tomoo Yamazaki, Michiharu Komatsu, Yoshihiko Katsuyama, Kaname Yoshizawa, Eiji Tanaka, Masao Ota

**Affiliations:** 1 Department of Medicine, Division of Hepatology and Gastroenterology, Shinshu University School of Medicine, Matsumoto, Japan; 2 Department of Pharmacy, Shinshu University Hospital, Matsumoto, Japan; 3 Department of Gastroenterology, NHO Shinshu Ueda Medical Center, Ueda, Japan; 4 Department of Legal Medicine, Shinshu University School of Medicine, Matsumoto, Japan

## Abstract

Autoimmune hepatitis (AIH) and primary biliary cholangitis (PBC) are liver-specific autoimmune conditions that are characterized by chronic hepatic damage and often lead to cirrhosis and hepatic failure. Specifically, the *protein tyrosine phosphatase N22* (*PTPN22*) gene encodes the lymphoid protein tyrosine phosphatase, which acts as a negative regulator of T-cell receptor signaling. A missense single nucleotide polymorphism (SNP) (rs2476601) in *PTPN22* has been linked to numerous autoimmune diseases in Caucasians. In the present series, nine SNPs in the *PTPN22* gene were analyzed in 166 patients with AIH, 262 patients with PBC, and 322 healthy controls in the Japanese population using TaqMan assays. Although the functional rs3996649 and rs2476601 were non-polymorphic in all subject groups, the frequencies of the minor alleles at rs1217412, rs1217388, rs1217407, and rs2488458 were significantly decreased in AIH patients as compared with controls (all *Pc* < 0.05). There were no significant relationships with PTPN22 SNPs in PBC patients. Interestingly, the AAGTCCC haplotype was significantly associated with resistance to both AIH (odds ratio [OR] = 0.58, *P* = 0.0067) and PBC (OR = 0.58, *P* = 0.0048). SNPs in the *PTPN22* gene may therefore play key roles in the genetic resistance to autoimmune liver disease in the Japanese.

Autoimmune diseases are characterized by an aberrant immune response to self-antigens. Although genetic factors contribute to disease susceptibility and severity, the mechanisms of disease initiation and persistence remain poorly understood. Autoimmune hepatitis (AIH)^1^,^2^ and primary biliary cholangitis (PBC)^3^,^4^ are prominent autoimmune diseases of the liver. Mutations in the human leukocyte antigen (HLA) region have been implicated in multiple autoimmune diseases, among which the HLA-DRB1^*^04:05-DQB1^*^04:01 and HLA-DRB1^*^08:03-DQB1^*^06:01 haplotypes were linked with susceptibility to AIH^5^,^6^ and PBC^7^ in Japanese populations. Polymorphisms of *cytotoxic T-lymphocyte antigen 4* have also been identified as susceptibility determinants in PBC^8^, but not in AIH^9^. Furthermore, there is overlap among polymorphic loci identified by genome-wide association studies and linkage studies in several autoimmune diseases between Caucasians and the Japanese^10^,^11^,^12^,^13^.

The *protein tyrosine phosphatase N22* gene (*PTPN22*) located on chromosome 1p13.3-13.1 encodes a lymphoid-specific protein tyrosine phosphatase (Lyp) that is important in the negative control of T-cell activation and in T-cell development. A missense single nucleotide polymorphism (SNP) known as rs2476601 in *PTPN22* has been consistently associated with a variety of autoimmune diseases in populations of European ancestry, including rheumatoid arthritis (RA), type I diabetes, and systemic lupus erythematosus (SLE)^14^,^15^,^16^,^17^,^18^, but this functional SNP was non-polymorphic and no relationships were found in studies from Japan^19^,^20^,^21^,^22^.

Genome-wide association studies have confirmed that *PTPN22* is associated with RA and type 1 diabetes^23^,^24^,^25^. Two SNPs, rs2488457 in the promoter region of *PTPN22* and rs1217412 in the 3′-untranslated region, have also been linked to the onset of acute type 1 diabetes in Japanese and Korean populations^18^, and another amino acid substitution, rs33996649 within the catalytic domain of the enzyme, was related to the development of autoimmune diseases^26^. Meanwhile, several studies have implicated *PTPN22* SNPs with autoimmune disorders independently of rs2488457, suggesting that such polymorphisms play a general role in autoimmunity. We therefore hypothesized that *PTPN22* SNPs may also be associated with autoimmune liver disease and investigated for relationships between *PTPN22* SNPs and AIH or PBC in Japanese patients.

## Results

### *PTPN22* Genotyping in Patients and Controls

The functional rs3996649 and rs2476601 SNPs were not polymorphic in any study group, which was in agreement with previous studies^19^,^20^,^21^,^22^, and were therefore excluded from further analysis. The genotype frequencies of the remaining seven tested *PTPN22* gene SNPs were in Hardy-Weinberg equilibrium in patients with AIH or PBC and in controls.

The minor allele frequencies at rs1217412, rs1217388, rs1217407, rs3765598, rs2488458, rs3789612, and rs2488457 were all significantly decreased among AIH patients as compared with healthy subjects (Table 1). Genotype frequencies also differed significantly between AIH patients and controls for rs1217412, rs1217388, rs1217407, rs3765598, rs2488458, and rs2488457 by a dominant model of inheritance (Table 2). The statistical power of this study was high at 0.999 when calculated as α = 0.05, β = 0.95, and sample number = 488.

The frequency of the minor T allele at rs3765598 was decreased in PBC patients as compared with healthy subjects (Table 1), as was positivity for the major C allele (CT+TT) at rs3765598 and rs3789612, but this difference was not significant after correction for multiple testing (Table 2).

### *PTPN22* Haplotypes in Patients and Controls

Pairwise linkage disequilibrium (LD) mapping confirmed that the seven tested alleles were in strong LD over a narrow range, with an LD index >0.9 (Table 3). Strong LD was indicated in the same block for AIH and PBC patients and controls. Nine unique SNP haplotypes were found altogether, of which four had frequencies of >5% (Table 4). Association analysis of haplotypes calculated by EM algorithms showed that haplotype 3, which was the only rs3765598 T, was significantly associated with resistance to both AIH (OR = 0.58, 95% CI 0.39–0.86, *P* = 0.0067) and PBC (OR = 0.58, 95% CI 0.40–0.85, *P* = 0.0048).

### Association Between *PTPN22* SNPs and Clinical Outcome

AIH and PBC patients were stratified according to disease progression. However, no SNP or haplotype in the *PTPN22* gene was associated with either cirrhosis in AIH or a history of orthotopic transplantation and disease progression of PBC (data not shown).

## Discussion

In recent studies on the *PTPN22* gene, the rs2476601 missense substitution SNP has been associated with multiple autoimmune diseases in Caucasians, including RA, SLE, Graves’ disease, and Addison’s disease. rs2476601 was also proposed to be functionally involved in interactions between Lyp and Csk kinase^13^,^14^,^27^. Another functional SNP that is located in the catalytic domain of Lyp, rs33996649, leads to reduced phosphatase activity and has been highlighted as an important genetic risk factor for RA and SLE^26^. The present analysis showed that both of these SNPs were non-polymorphic (rs2476601: C, rs33996649: G) among all subject groups, in accordance with earlier studies from Asia^19^,^20^,^21^,^22^. Our data confirmed that the rs2476601 and rs33996649 SNPs were not associated with Japanese autoimmune liver disease.

**Figure 1:**
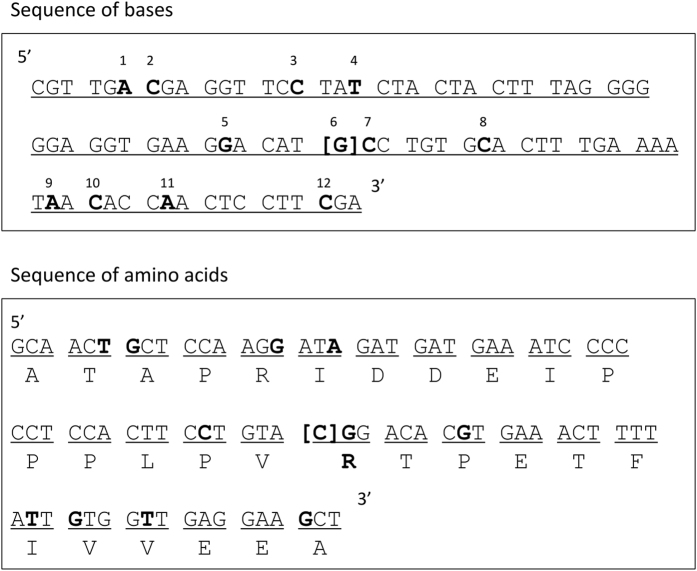
Alignments of Bases (**A**) and Amino Acids (**B**) of Exon 14 in *PTPN22*. SNPs in bold type are polymorphic. 1; rs554195846 (A/G: Thr/Thr), 2; rs138223016 (C/A: Ala/Ser), 3; rs765535869 (C/T: Arg/Arg), 4; rs759881801 (T/C: Ile/Met), 5; rs764275349 (G/A: Pro/Leu), 6; rs2476601 (G/T: Arg/Trp), 7; rs201811041 (C/T: Arg/Gln), 8; rs74163660 (C/G: Pro/Arg), 9; rs768160390 (A/G: Ire/Thr), 10; rs368086285 (C/T: Val/Met), 11; rs775140391 (A/G: Val/Ala), 12; rs569454620 (C/T: Ala/Thr).

This study revealed a striking association between SNPs in the *PTPN22* gene and resistance to AIH. Although a genome-wide association study showed that *PTPN22* was not related to AIH in patients of European descent^13^, there have been no data regarding *PTPN22* SNPs in Japanese AIH. Hence, our results raise several possibilities on an association between *PTPN22* loci and AIH protection in the Japanese population. First, among the five significantly associated SNPs in *PTPN22*, rs2488457 in the promoter region might be an important factor in Asian populations, as seen in case-control studies where it increased the risk of RA^28^,^29^ and ankylosing spondylitis^30^. Second, other potentially functional variants may be engaged in susceptibility to AIH as there are nine non-synonymous substitutions in addition to rs2476601 in exon 14 of *PTPN22*. Lastly, it is possible that the *PTPN22* locus contains another, undefined functional variant in LD with rs3996649 or rs2476601. To address these prospects, we sequenced exon 14 of *PTPN22* in 12 patients with AIH and 12 healthy controls whose genotypes were GG, AG, and AA at rs1217388 adjacent to rs2476601. No missense substitutions were detected in any sample, and all alleles were wild type (Fig. 1). However, the strong LD across this region as evidenced by pairwise D’ values near 1 (Table 3) made it difficult to ascertain whether these associated SNPs were independent protective factors of AIH. We compared haplotype frequencies between patients and controls to address this problem. Haplotype 3 containing AAGTCCC was less frequent in AIH and significantly associated with disease resistance (*P* = 0.0067, OR = 0.58). This novel haplotype contained minor and protective alleles concerning AIH susceptibility.

Although various autoimmune disorders have been associated with rs2476601, negligible relationships were found for systemic sclerosis, celiac disease, ulcerative colitis, Crohn’s disease, multiple sclerosis, and psoriasis in a meta-analysis^31^. The study defined two groups of diseases with regard to their targeted tissues, and showed that most autoimmune diseases possessing an insignificant association with rs2476601 manifested in the skin, gastrointestinal tract, bile duct, or immune privileged sites. Such results indicated that the relationship of individual *PTPN22* SNPs with autoimmune diseases depended on the localization of the affected tissues and suggested a role of targeted organ variation in disease manifestations. Regarding autoimmune liver disease, only one association study on PBC has been conducted that showed no relationship with rs2476601 in Canada^32^. No genome-wide association studies have demonstrated a link between *PTPN22* and PBC susceptibility^10^,^11^,^12^,^33^,^34^, which was supported by our data. PBC is characterized by slow, progressive destruction of the small bile ducts within the liver. Primary sclerosing cholangitis is another autoimmune disease targeting the bile ducts that is complicated by ulcerative colitis. Primary sclerosing cholangitis was also not associated with SNPs in rs2476601^35^. Interestingly, we witnessed that haplotypes containing the rs3765598 T allele were significantly associated with a 0.6 times less likelihood to develop PBC, which suggested that this haplotype may play an important role in protection from PBC in Japan. The limitations of this study are a small number of cases and controls and a narrow focus on few SNPs in this era of genome-wide association studies. Further investigation is needed to validate this association in other Asian countries.

In conclusion, the present study revealed *PTPN22* gene SNP and haplotype associations with protection against AIH or PBC in a Japanese population. This gene may therefore play a crucial role in the pathogenesis of Japanese autoimmune liver disease, and further studies are warranted to clarify its role in AIH and PBC.

## Patients and Methods

### Subjects

The clinical and biochemical features of the 166 patients with AIH and 262 patients with PBC enrolled in this study between January 2001 and August 2015 are summarized in Table 5. We also recruited 322 volunteer control subjects from hospital staff who had indicated the absence of any major illnesses in a standard questionnaire. The racial background of all individuals was Japanese. All AIH patients had been diagnosed according to the scoring system of the International Autoimmune Hepatitis Group^36^ and were classified as having type 1 AIH based on antibody profiles. The diagnosis of PBC was made according to criteria from the American Association for the Study of Liver Diseases^37^. Anti-nuclear antibody titers were determined by immunofluorescence using HEp-2 cells, for which a titer of ≥1:80 was considered a positive result^38^. Anti-mitochondrial antibody-M2 was measured by the enzyme-linked immunosorbent assay as reported previously^8^. All patients were negative for the hepatitis B surface antigen and antibodies to the hepatitis B core antigen, hepatitis C virus, and human immunodeficiency virus. Overlap syndromes were excluded. Liver cirrhosis was diagnosed by histological examination and/or characteristic clinical signs of advanced liver disease^39^. This study was reviewed and approved by the Institutional Review Board of Shinshu University Hospital (Matsumoto, Japan), and written informed consent was obtained from all participating subjects. The investigation was conducted according to the principals of the Declaration of Helsinki.

### *PTPN22* Genotyping

Genomic DNA from patients and controls was isolated from whole blood samples using QuickGene-800 assays (Fujifilm, Tokyo, Japan).

We evaluated nine SNPs (rs1217412, rs1217388, rs2476601, rs1217407, rs3765598, rs33996649, rs2488458, rs3789612, and rs2488457) spanning a 58 kb region in the *PTPN22* gene. The SNPs were selected from previous reports^14^,^15^,^19^,^22^ and had minor allele frequencies of >5% according to HapMap Japanese data (http://hapmap.ncbi.nlm.nih.gov/). Genotyping of all SNPs was performed with a TaqMan 5′ exonuclease assay using primers supplied by Applied Biosystems (Foster City, CA, USA). The probe’s fluorescence signals were detected with the StepOne Plus Real-Time PCR System (Applied Biosystems) according to the manufacturer’s instructions.

### Statistical Analysis

Allele, genotype, and haplotype frequencies along with Hardy-Weinberg equilibrium and LD were assessed using SNPStats software (Catalan Institute Oncology, Barcelona, Spain; http://bioinfo.iconcologia.net/SNPstats)^40^ and Haploview 4.1 software^41^. For analysis of genotype data, we adopted the multiple inheritances model to assess each minor allele, including codominant 1 (AB vs. BB, assuming that A is the minor allele), codominant 2 (AA vs. BB), dominant (AA+AB vs. BB), recessive (AA vs. AB+BB), over-dominant (AB vs. AA+BB), and log-additive (AA vs. AB vs. BB) models. Akaike’s information criterion was used to determine the most suitable inheritance model^42^. *P* values were subjected to Bonferroni correction by multiplication by the number of different SNPs. A *P* value of less than 0.05 was considered to be statistically significant.

## Additional Information

**How to cite this article**: Umemura, T. *et al*. Genetic Association of *PTPN22* Polymorphisms with Autoimmune Hepatitis and Primary Biliary Cholangitis in Japan. *Sci. Rep.* **6**, 29770; doi: 10.1038/srep29770 (2016).

## Figures and Tables

**Table 1:** Allelic Association Tests of Seven Genotyped SNPs in *PTPN22*.

SNP	Position on Chr. 13 (Build 37.p13)	Gene location	Alleles (1 > 2)	Minor allele frequency, %			Controls vs. AIH			Controls vs. PBC	
				Controls(n = 322)	AIH(n = 166)	PBC(n = 262)	*P* value	*Pc* value	OR (95% CI)	*P* value	*Pc* value
rs1217412	113814589	3′UTR	G>A	42.4	33.4	42.6	0.0066	**0.046**	0.68 (0.52–0.90)	0.954	>1.0
rs1217388	113821854	intron	G>A	42.4	33.4	42.6	0.0066	**0.046**	0.68 (0.52–0.90)	0.954	>1.0
rs1217407	113851126	intron	A>G	42.4	33.2	42.6	0.0066	**0.046**	0.68 (0.52–0.90)	0.954	>1.0
rs3765598	113851841	intron	C>T	21.6	15.1	16.4	0.0145	0.087	0.64 (0.45–0.92)	0.026	0.18
rs2488458	113863829	intron	T>C	42.6	33.4	42.6	0.0066	**0.046**	0.68 (0.52–0.90)	0.954	>1.0
rs3789612	113871486	intron	C>T	12.4	7.8	5.7	0.0400	0.24	0.60 (0.37–0.98)	0.065	0.46
rs2488457	113872746	5′ near gene	G>C	42.2	33.4	38.2	0.0076	0.053	0.69 (0.52–0.91)	0.159	>1.0

1, major allele; 2, minor allele; OR, odds ratio; CI, confidence interval.

**Table 2:** Genotype Distribution of *PTPN22* Gene Polymorphisms in Patients with AIH or PBC and Healthy Controls.

SNP	Alleles	Genotype	Genotype frequency,%			Model^*^	Controls vs. AIH			Controls vs. PBC		
	(1>2)		Controls	AIH	PBC		*P* value	*Pc* value	OR (95% CI)	*P*value	*Pc* value	OR (95% CI)
			(n = 322)	(n = 166)	(n = 262)							
rs1217412	G>A	AA/AG/GG	15.2/54.4/30.4	10.2/46.4/43.4	17.9/49.2/32.8	Dominant	0.0048	**0.034**	0.57 (0.39–0.84)	0.54	>1.0	
						(AA+AG vs.GG)						
rs1217388	G>A	AA/AG/GG	15.2/54.4/30.4	10.2/46.4/43.4	17.9/49.2/32.8	Dominant	0.0048	**0.034**	0.57 (0.39–0.84)	0.54	>1.0	
						(AA+AG vs.GG)						
rs1217407	A>G	GG/GA/AA	15.2/54.4/30.4	10.2/46.4/43.4	17.9/49.2/32.8	Dominant	0.0048	**0.034**	0.57 (0.39–0.84)	0.54	>1.0	
						(GG+GA vs. AA)						
rs3765598	C>T	TT/TC/CC	3.1/37.0/59.9	1.8/26.5/71.7	3.0/26.7/70.2	Dominant	0.0097	0.068	0.59 (0.39–0.89)	0.0095	0.067	0.63 (0.45–0.90)
						(TT+TC vs. CC)						
rs2488458	T>C	CC/CT/TT	15.2/54.4/30.4	10.2/46.4/43.4	17.9/49.2/32.8	Dominant	0.0048	**0.034**	0.57 (0.39–0.84)	0.54	>1.0	
						(CC+CT vs. CC)						
rs3789612	C>T	TT/TC/CC	0.6/15.8/83.5	0.0/15.7/84.3	1.1/9.2/89.7	Dominant	0.82	>0.2		0.03	0.21	0.58 (0.36–0.96)
						(TT+TC vs. CC)						
rs2488457	G>C	CC/CG/GG	14.9/54.7/30.4	9.6/47.6/42.8	13.4/49.6/37.0	Dominant	0.007	**0.049**	0.59 (0.40–0.86)	0.094	0.66	
						(CC+CG vs. CC)						

1, major allele; 2, minor allele; OR, odds ratio; CI, confidence interval.

^*^ The model with the smallest Akaike’s information criterion value was defined as the most appropriate model for each SNP.

**Table 3:** Pairwise LD of Seven SNPs in *PTPN22* Among 488 Patients with AIH and Healthy Controls.

	rs1217412	rs1217388	rs1217407	rs3765598	rs2488458	rs3789612	rs2488457
rs1217412	—	1.000	1.000	0.989	1.000	0.933	0.974
		1.000	1.000	0.361	1.000	0.051	0.945
rs1217388		—	1.000	0.989	1.000	0.933	0.974
			1.000	0.362	1.000	0.051	0.945
rs1217407			—	0.989	1.000	0.933	0.974
				0.361	1.000	0.050	0.945
rs3765598				—	0.989	1.000	0.944
					0.361	1.000	0.331
rs2488458					—	0.933	0.974
						0.050	0.945
rs3789612						—	1.000
							0.021
rs2488457							—

The degree of LD is shown as a measure of D’ (upper) and r^2^ (lower) in each column.

**Table 4:** Estimated Haplotype Frequencies of *PTPN22* Gene Polymorphisms in Patients with AIH or PBC and Healthy Controls.

	rs1217412	rs1217388	rs1217407	rs3765598	rs2488458	rs3789612	rs2488457	Frequency, %			Controls vs. AIH		Controls vs. PBC	
								Controls(2n = 644)	AIH(2n = 332)	PBC(2n = 524)	*P* value	OR (95% CI)	*P* value	OR (95% CI)
1	G	G	A	C	T	C	G	48.7	58.4	52.3		1.00		1.00
2	A	A	G	C	C	C	C	21.0	18.4	24.8	0.059	0.70(0.48–1.01)	0.74	1.05(0.78–1.41)
3	A	A	G	T	C	C	C	20.1	14.8	12.5	**0.0067**	0.58(0.39–0.86)	**0.0048**	0.58(0.40–0.85)
4	G	G	A	C	T	T	G	7.9	7.8	4.3	0.37	0.79(0.47–1.33)	0.062	0.59(0.33–1.03)

Abbreviations: OR, odds ratio; CI, confidence interval.

**Table 5:** Demographic and Clinical Characteristics of Patients with AIH or PBC.

Characteristic	AIH (n = 166)	PBC (n = 262)
Median age, years (range)	59 (22–87)	58 (27–86)
Female, n (%)	147 (89)	234 (89)
Cirrhosis, n (%)	18 (11)	45 (17)
OLT, n (%)	0 (0)	12 (5)
Serum ANA-positive, n (%)	159 (96)	215 (82)
Serum AMA-positive, n (%)	0 (0)	238 (91)

Abbreviations: AIH, autoimmune hepatitis; PBC, primary biliary cirrhosis; OLT, orthotopic liver transplantation; ANA, anti-nuclear antibody; AMA, anti-mitochondrial antibody.
